# Effects of confined thermal cycling on sealants with different thermomechanical properties for CCS

**DOI:** 10.1038/s41598-025-19942-3

**Published:** 2025-09-17

**Authors:** Kai Li, Reinier van Noort, Anne Pluymakers

**Affiliations:** 1 Geoscience and Engineering Department, Delft University of Technology, Delft, The Netherlands; 2 Geothermal Energy and Reservoir Technology, Karlsruhe Institute of Technology, Karlsruhe, Germany; 3 Institute for Energy Technology (IFE), Kjeller, Norway; 4 Institute of Soil Mechanics and Rock Mechanics (IBF), Engler-Bunte-Ring 14, 76131 Karlsruhe, Germany

**Keywords:** CCS, Sealant integrity, Thermomechanical properties, Thermal cycling, Confinement

## Abstract

In geological CO_2_ storage, the integrity of seals between well and caprock is crucial for ensuring permanent CO_2_ storage. One key mechanism that might affect this integrity is thermal cycling when cold CO_2_ is injected periodically into a hot reservoir. Our study aims to identify which properties of a sealant are key to its ability to withstand thermal cycling under in-situ conditions. We investigate sealants of four different compositions, namely two ordinary Portland cement-based blends (S1 and S2), and two blends designed for carbon capture and storage applications, namely a novel ordinary Portland cement-based blend with CO_2_-sequestering additives (S3), and a calcium aluminate cement-based blend (S4). These four sealants possess different thermomechanical properties, such as Young’s modulus, tensile strength, unconfined compressive strength, thermal diffusivity, and thermal expansion coefficient, also characterized in this study. Samples of these sealants were placed in a triaxial deformation apparatus, and subjected to either 1.5 or 10 MPa confinement at 120 °C. Then we applied eight thermal cycles by injecting 20 °C water through a central bore in these samples. To assess the effects of the thermal treatment, we used X-ray micro-computed tomography (micro-CT), helium pycnometry, and compressive strength testing on thermal-treated as well as intact samples. Micro-CT results indicate that all sealant samples maintained integrity without cracking (above 32 µm) after thermal cycling under confinement. For all four sealants, post-treatment porosity (determined by either micro-CT or pycnometer) was reduced, which is ascribed to compression during confinement. This reduction in porosity was associated with an increase in compressive strength. Compared to experiments conducted under 1.5 MPa confinement, those at 10 MPa exhibit a greater reduction in porosity, and more enhanced compressive strength. The application of confinement suppressed the potential of crack formation by increasing the effective strength including tensile strength of the sealant during thermal cycling by a reduction in porosity. Based on these findings, we conclude that to limit potential damage to seal integrity induced by thermal cycling, sealants for carbon capture and storage should ideally have high tensile strength and thermal diffusivity, but low Young’s modulus and thermal expansion coefficient.

## Introduction

Carbon capture and storage (CCS) technology has gained significant attention as a promising approach to mitigate global climate change to store CO_2_ in subsurface formations, such as saline aquifers or former hydrocarbon reservoirs^1–5^. In order to be successful, CO_2_ needs to be permanently stored in the subsurface without leakage. Sites are usually screened beforehand to ensure minimum risk of leakage, but that doesn’t account for potential damage which may occur during the injection period. Reservoirs are usually at 2–4 km depth, where temperatures typically range from 80 to 120 °C. If CO_2_ injection occurs into offshore reservoirs (for example the Dutch Portos project or the Norwegian Northern Lights project), the injected CO_2_ may have temperature as low as 0 °C (temperature of the sea floor). This can lead to temperature fluctuations of up to 100 °C during periodic injection, resulting in cyclic shrinkage and subsequent expansion of the wellbore and subsurface formations^6–8^. Consequently, cracks may form in the cement and between casing, cement sheath, and surrounding wall-rock. These cracks potentially lead to micro-annuli (microscopic gaps or channels that can form around the casing or cement sheath, which can significantly affect well integrity), which could pose a significant challenge to the permanent geological storage of CO_2_^9–12^. Moreover, injection is expected to occur periodically, so cold pressurized CO_2_ is injected into warm reservoirs, meaning fluctuations of reservoir temperature will be cyclic, where the precise intensity, rate, and frequency are uncertain^6,8,13,14^.

Ordinary Portland cement (OPC) is commonly used as the main component in sealants in oil and gas wells, due to its reliable performance and cost-effectiveness^15–17^, and is therefore expected to be common in legacy wells that may penetrate depleted reservoirs targeted for geological CO_2_ storage. However, OPC is relatively vulnerable to chemical deterioration when exposed to water and CO_2_^17,18^. To mitigate the risk associated with CO_2_ storage, novel blends are being designed. Such blends may be based on OPC but with enhanced ability to withstand exposure to CO_2_, through reducing permeability or by adding crushed dunite (mainly olivine) to alter chemical behaviour^19,20^. Alternatively, CO_2_-resistance may be improved through using a different cementing system altogether, such as those based on calcium aluminate cement (CAC). It offers advantages over OPC, such as higher early strength gain and enhanced acid resistance^21,22^, making it attractive for CCS applications. Previous research on thermal-cycling damage has primarily focused on the integrity of the interfaces between the casing, cement sheath, and surrounding rock, and has been largely limited to studies involving ‘standard’ OPC-blends^23–25^.

The effects of thermal cycling on the integrity of the sealant material itself for CCS applications have remained largely unexplored. A recent contribution to this area is the work of Li and Pluymakers ^26^, who investigated the sealing integrity of the same four sealant compositions as tested here under unconfined conditions, with a similar thermal shock protocol with relevance to CCS applications. Their unconfined tests revealed a critical difference in performance of the four sealants: only the novel OPC-based blend with CO_2_-sequestering additives maintained its integrity, whereas the two conventional OPC blends and the calcium aluminate cement-based blend all suffered cracking. This highlights the vulnerability of most sealants to thermal cycling in the absence of confining pressure. However, actual CO_2_ storage environments occur at depth^13,27,28^, where at 1 km the wellbore including sealant sheath would already experience confining pressures up to 10 MPa. The presence of this confinement is expected to mitigate or prevent the formation of new microcracks due to thermal stresses.

To understand how confinement may act to limit damage to sealants when exposed to thermal cycles, we perform experiments on four sealants placed under confining pressures of 1.5 and 10 MPa, at a temperature of 120 °C. We use cylindrical sealant samples with a central borehole and flow cold water through hot samples to perform the thermal cycles. The temperatures of the sample and injected water, and the rate and the duration of the injection are the same as for the flow-through experiments in Li and Pluymakers^26^. Microstructural scanning, pycnometry and mechanical tests are carried out before and after experiments to examine the effects of thermal cycling on sealants under confinement. Note that in the current study, we perform a more extensive thermomechanical characterization of bulk properties than previously done by Li and Pluymakers^26^, as we also determine tensile strength, linear thermal expansion coefficient and porosity, on top of the known Young’s modulus, unconfined compressive strength and thermal diffusivity. By knowing the entire suite of thermomechanical properties, we can more accurately estimate the efficacy of a sealant to tolerate thermal stresses and maintain its integrity in CCS. We aim to identify the key properties of a sealant that determine its performance through strong thermal cycling under pressure and temperature conditions representative for a depth of 2–4 km, thereby contributing to the advancement of wellbore sealant design and testing for CCS applications.

## Experimental materials, apparatus, and methodologies

### Sample description

In our study, we employ cylindrical sealant samples (diameter 3 cm, length 7 cm) with a central borehole (4 mm diameter) parallel to the vertical axis. The samples represent four different compositions, as outlined in Table 1, labelled S1 to S4 (Table 1). As can be seen in Table 1, S1 and S2 are more conventional OPC-based blends, where S2 is an ultra-low permeability blend for field design. S1 and S2 would be expected to be found in legacy wells in former oil and gas reservoirs that are now targeted for CCS. S3 and S4 are more novel, with S3 being an OPC-based blend with added olivine (added as crushed dunite). The added olivine is expected to improve resistance of this blend to exposure to CO_2_, through olivine’s reaction with CO_2_ to form MgCO_3_^29^. Due to its relatively low binder content, S3 has different mechanical properties than more conventional OPC-blends. S4 is a CAC-based blend, which is highly acid resistant and is tailored specifically for CO_2_ storage environment.

**Table 1:** An overview of the four sealant compositions studied here, with their Specific Gravities (SG).

Sealant	Composition
S1	1.90 SG class G cement with 35% BWOC silica flour
S2	1.90 SG ultra-reduced permeability class G cement with 35% BWOC silica flour, with high silica fume concentration and expansion agent in form of dead-burnt MgO
S3	1.90 SG composition based on S2, replacing 28.5% of the binder with olivine-based CO_2_-sequestering agent
S4	1.80 SG calcium aluminate-based blend

Note that the same table is found in Li and Pluymakers^26^, given that both studies worked with the same sample batch.

The samples used are cast and cured by Halliburton AS Norway, following API Recommended Practice 10B-2 (API RP 10B-2^30^). Samples are mixed with a water/cement ratio of 0.4, and cured at 150 °C and 30 MPa for 28 days. Thanks to the elevated temperature and pressure during curing, the hydration reactions would run to (near-)completion during this curing period. And subsequent changes in mechanical and thermal properties of the samples during storage and testing would be negligible. After curing, all samples are stored in fresh water at room temperature and pressures throughout the study period. Before use, the samples are dried in an air-circulated oven (model UF75, Memmert) for 2 days at 80 °C (ramping rate of 2.5 °C/min). This means the samples are 100% dry (confirmed by no further weight reduction after 2 days). Note that the slow temperature ramping rate minimizes the damage induced by the drying process, since thermal stresses that built up during heating and cooling have time to dissipate instead of leading to mechanical damage. While wellbore seals under in-situ conditions would likely contain some pore water, the use of dried samples is a requirement in this study, to avoid any impact of variable water content on sample behaviour, and isolate the thermomechanical properties of the sealant materials themselves.

### Pre- and post-thermal cycling thermomechanical analyses

Before commencing any thermal-cycling experiments, we perform additional Brazilian disc tests (50 kN loading frame) on dried disc sealant samples (diameter 3 cm, thickness 1.5 cm) cut from untreated samples to measure the tensile strength, $\sigma_{T}$. Displacement is controlled by two high-precision linear variable differential transformers (LVDTs; a 2 mm range and displacement rate of 0.0005 mm/s). The equation to calculate tensile strength is given by ASTM E8/E8M-16a^31^:

1 $$\sigma_{t} = \frac{2 P}{{\pi D L}}$$

where $P$ is the load at which the sample fails, $D$ the sample diameter, and $L$ the thickness of the disc sample.

In addition, we use a thermomechanical analyzer (PerkinElmer, TMA 4000) to measure the linear thermal expansion coefficient and a helium gas pycnometer (Anton Paar, Ultrapyc 5000) to measure the effective porosity of the dried sealants. Table 2 provides the results of our measurements on tensile strength, thermal expansion coefficient, and effective porosity of all samples post-curing and post-drying. To ensure that the obtained results are not unduly influenced by an ongoing sealant curing process, i.e., to verify that the curing procedure performed by Halliburton in sample preparation was sufficient to achieve a thermomechanical equilibrium, tensile strength, thermal expansion coefficient, and effective porosity are averaged for measurements on three samples each. These three measurements are conducted at monthly intervals over a period of 3 months. The small standard deviations show that our measurements are repeatable, and properties of sealants have not changed throughout our study duration. Note that other mechanical properties (unconfined compression strength (UCS), Young’s modulus, and Poisson’s ratio), bulk density, and thermal properties (thermal conductivity, specific heat capacity, and thermal diffusivity) that were previously reported by Li and Pluymakers^26^ are also provided here in Table 2.

**Table 2:** Properties of the four sealants, as measured before thermal treatment but after drying (^*^ properties of sealants that were determined by Li and Pluymakers^26^).

Sealant	Tensile Strength [MPa]	Linear thermal expansion coefficient [10^−6^ °C^−1^]	Effective porosity by helium pycnometer [%]	Unconfined compressive strength [MPa]*	Young’s modulus [GPa]*	Poisson’s ratio [-]*	Bulk density [kg/m^3^]*	Thermal conductivity [W/(m K)]*	Specific heat capacity [J/(kg K)]*	Thermal diffusivity [mm^2^/s]*
S1	3.63 ± 0.35	6.89 ± 0.20	35.6 ± 1.9	99.8 ± 1.1	13.4 ± 0.2	0.143 ± 0.005	1455	0.82 ± 0.04	878 ± 18	0.640
S2	5.51 ± 0.32	10.93 ± 0.62	26.8 ± 2.1	81.1 ± 1.3	12.0 ± 0.2	0.162 ± 0.010	1507	0.93 ± 0.03	936 ± 11	0.660
S3	6.79 ± 0.15	9.40 ± 0.12	42.2 ± 0.9	33.4 ± 0.7	6.1 ± 0.1	0.139 ± 0.009	1374	1.04 ± 0.02	684 ± 13	1.110
S4	3.92 ± 0.10	12.32 ± 0.53	37.5 ± 1.6	34.3 ± 1.3	6.6 ± 0.1	0.172 ± 0.014	1497	0.89 ± 0.02	970 ± 21	0.610

To determine how thermal cycling under confinement affects the integrity of our sealants, we scan all samples before and after thermal treatment using an X-ray micro-computed tomography scanner (Nanotom 180 NF, Phoenix X-ray Systems & Services GmbH; voxel resolution of 32 µm). Resulting images are post-processed with Phoenix datos software (version 2.0, GE Measurement & Control solutions) to compare how the 3D microstructure in samples changes during the experiments. Due to technical limitations, any voids (discrete, resolvable cavities) or cracks (thin, fracture-like features) smaller than 32 µm (the voxel size) cannot be detected. We then use Avizo software (version 2020.2, ThermoFisher Scientific) to quantify the volume of micro-cracks and voids, and the volume fractions of the samples attributed to these features, following the workflow outlined by Li and Pluymakers^26^. In addition, we measure post-experimental effective porosity and then the UCS after experiments to determine how thermal cycling affects these macroscopic properties. All samples and the experimental scheme are outlined in Table 3.

**Table 3:** Sample overview and a schematic of the experimental procedure.

No	Sample name	Sealant composition	Confining pressure [MPa]	Experiment scheme
1	S1H-1	S1, OPC blend	1.5	helium pycnometry ↓ micro-CT ↓ thermal cycling ↓ micro-CT ↓ helium pycnometry ↓ UCS
2	S1H-2		10	
3	S2H-1	S2, OPC blend with ultra-low permeability	1.5	
4	S2H-2		10	
5	S3H-1	S3, OPC blend with CO_2_-sequestering additives	1.5	
6	S3H-2		10	
7	S4H-1	S4, CAC blend	1.5	
8	S4H-2		10	

### Thermal cycling procedure

**Fig. 1:**
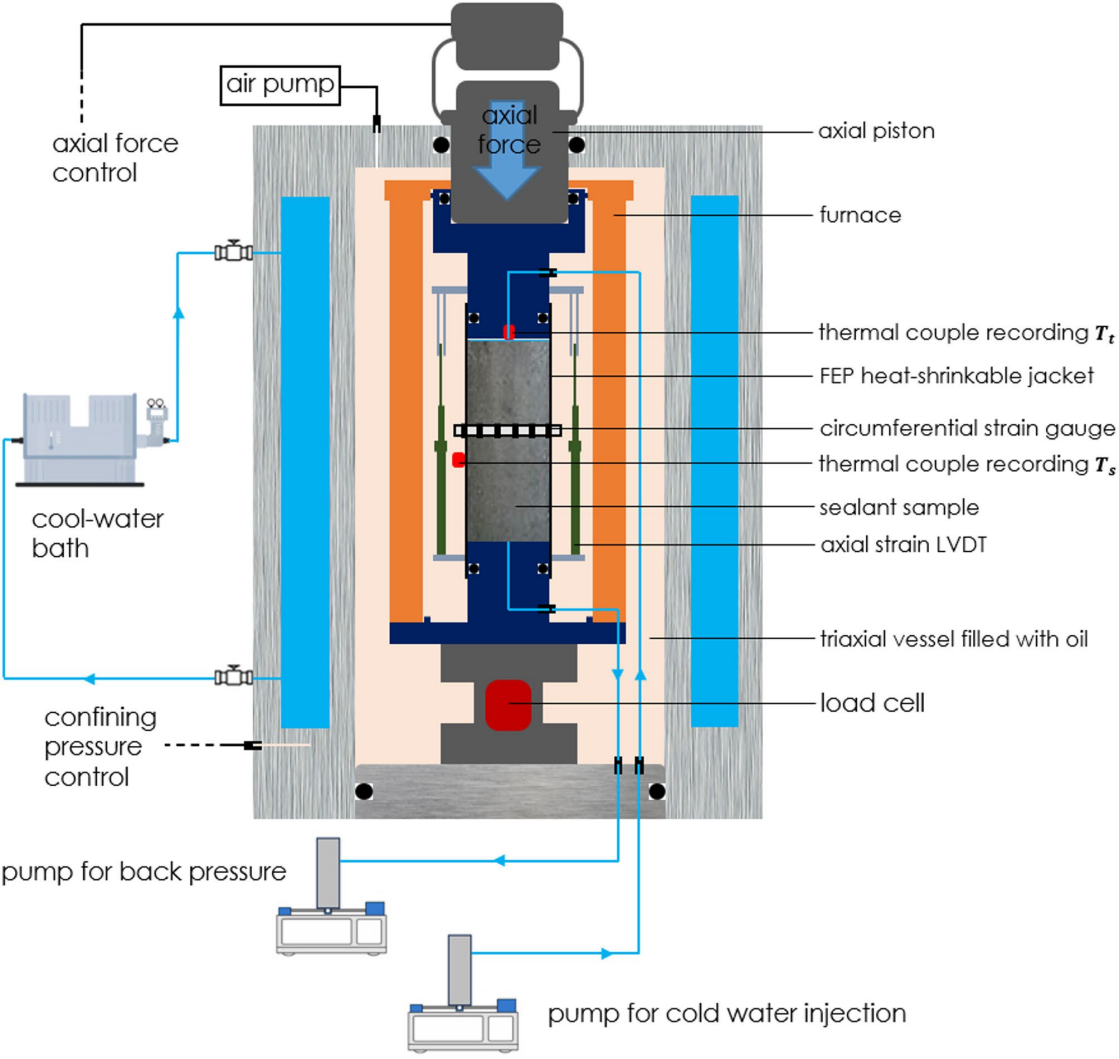
Schematic of the triaxial deformation apparatus with sealant sample mounted inside the pressure vessel. The drawing does not adhere to the actual scale.

To expose our samples to thermal cycling under a confining pressure, we use a standard triaxial deformation apparatus (Fig. 1) capable of fluid flow through a confined sample. The confining pressures in this apparatus can be maximum 70 MPa, and axial force can be maximum up to 300 kN (equivalent to 424 MPa axial stress on a cylindrical sample with a diameter of 3 cm). As shown in Fig. 1, an internal furnace is used to achieve an elevated temperature in the vessel. The sample, jacketed by a heat-shrinkable FEP (fluorinated ethylene propylene) tube (wall thickness 0.5 mm after shrinking), is mounted between the upper and lower axial pistons. In both pistons, pore fluid lines are fitted to allow water injection through the sample. During the experiments, the triaxial vessel is filled with heat transfer oil (Shell Thermia oil B) that provides the confining pressure and transmits the heat. Two LVDTs (2 mm range) mounted parallel to the sample, and one circumferential strain gauge (10 mm range) mounted around the sample are used to monitor axial and radial deformation, respectively. Two thermocouples (type K, NI-9219, National Instruments rated to 700 °C with an accuracy ± 1 °C) are installed to measure the temperature on the outer surface in the sample centre ($T_{s}$), as well as on the top of the sample ($T_{t}$). During thermal-cycling experiments, a flow of cooling water runs through the outer shell of the confinement vessel to cool and protect the electronics of the apparatus from elevated temperature.

**Fig. 2:**
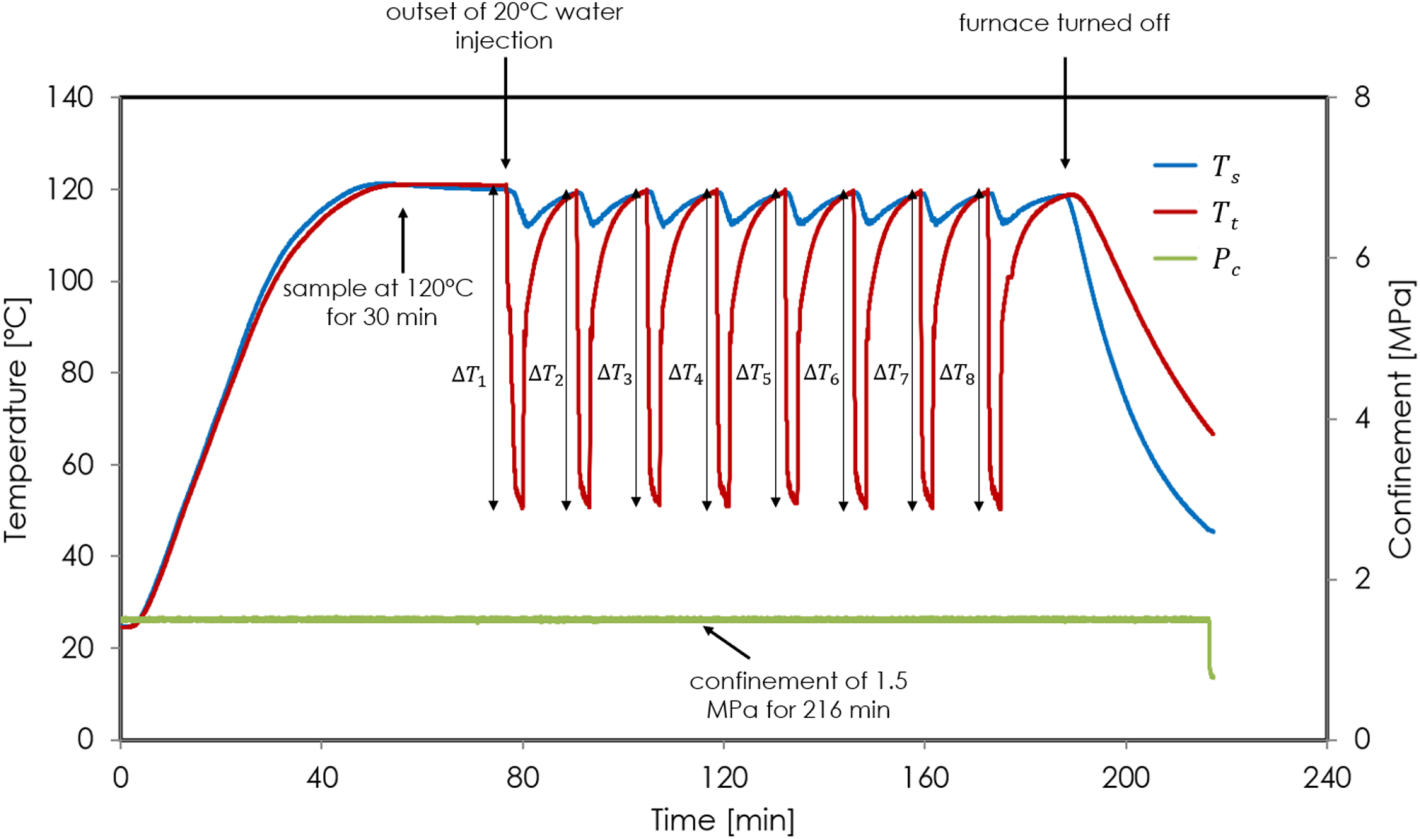
Temperature variations at the outer surface ($T_{s}$) and inlet ($T_{t}$) of sample S1H-1, and applied confinement ($P_{c}$) during thermal-cycling experiment. The thermal cycles shown here are representative for all experiments reported.

In thermal-cycling experiments, to mimic an in-situ state of stress, we load the sample at near hydrostatic conditions either at a confining pressure of 1.5 MPa with an axial stress of 4 MPa, or at a confining pressure of 10 MPa with an axial stress of 15 MPa. We then heat the pressure vessel filled with oil to 120 °C at a ramping rate of 2.2 °C/min, and maintain the system at this temperature for 30 min to allow the sample to be fully heated, as shown by both thermocouples (Fig. 2). We then inject 20 °C water from top to bottom through the sample using a high-pressure syringe pump (model 1000D, Teledyne ISCO, range: 0.001 to 408 mL/min, accuracy: 0.5% of setpoint) at 80 mL/min for 2 min, with a back pressure controlled by another syringe pump (model 260D, Teledyne ISCO, range: 0.001 to 107 mL/min, accuracy: 0.5% of setpoint). The back pressure is set at 0.5 MPa in experiments under 1.5 MPa confinement, and 6 MPa in experiments under 10 MPa confinement. Note that the reported confining pressure in our study is the effective confining pressure, defined as the absolute confining pressure minus the pore pressure (back pressure) maintained at the borehole outlet. After 2 min of injection, we stop for 12 min to let the system reheat the sample back to 120ºC, as shown by both thermocouples (Fig. 2). We repeat this for eight cycles. For each sealant composition, we test two samples, with one at 1.5 MPa confinement and one at 10 MPa confinement (Table 3).

Figure 2 illustrates the temperature variations at different positions of sample S1H-1, and the applied confinement, $P_{c}$, during the experiment. These changes serve as a representation of experiments conducted on all samples. As depicted, temperatures on the outer surface, $T_{s}$, and on the top (flow inlet), $T_{t}$, of the sample reach 120 °C following gradual heating by the furnace. These temperatures remain constant at 120 °C until the commencement of thermal cycling. Upon initial water injection, $T_{t}$ rapidly decreases to 50 °C within 2 min (at a rate of 35 °C/min), while $T_{s}$ decreases to 112 °C within the same timeframe. Subsequently, both temperatures equilibrate back to the system temperature at 120 °C, ready for the next injection cycle. This temperature fluctuation recurs eight times before the temperatures gradually decrease to room temperature upon shutting down the furnace. Throughout the whole process, sample S1H-1 is exposed to 1.5 MPa confinement. Note that we take care to ensure that the total duration each of our samples spend under confinement is similar, ranging from 212 to 224 min.

### Calculation of thermal stress and volumetric strain

As shown in Fig. 2, the most significant temperature fluctuation is observed near the sample inlet region, where the thermal cycling induces the highest thermal stress throughout the sample. We determine this maximum thermal stress, $\sigma_{T}$, using the following equation^32,33^:

2 $$\sigma_{T} = E \cdot \gamma \cdot \Delta T_{t}$$

where $E$ is Young’s modulus, $\gamma$ the thermal expansion coefficient of the sealant, and $\Delta T = \frac{{\mathop \sum \nolimits_{1}^{8} \Delta T_{{t_{i} }} }}{8}$ is the average temperature drop at the inlet of the sample within one injection cycle.

After thermal treatment, the samples are allowed to gradually return to room temperature, and then confinement and axial stress are released from the sample. We record the axial strain, $\varepsilon_{z}$, and circumferential strain, $\varepsilon_{\theta }$, of the sample, based on which we calculate the volumetric strain, $\varepsilon_{V}$, given by:

3 $$\varepsilon_{V} = 1 - \left( {1 - \varepsilon_{z} } \right) \cdot \left( {1 - \varepsilon_{\theta } } \right)^{2} \approx \varepsilon_{z} + 2\varepsilon_{\theta }$$

## Results

### Microstructure and porosities of sealant samples before and after thermal cycling under confinement

**Fig. 3:**
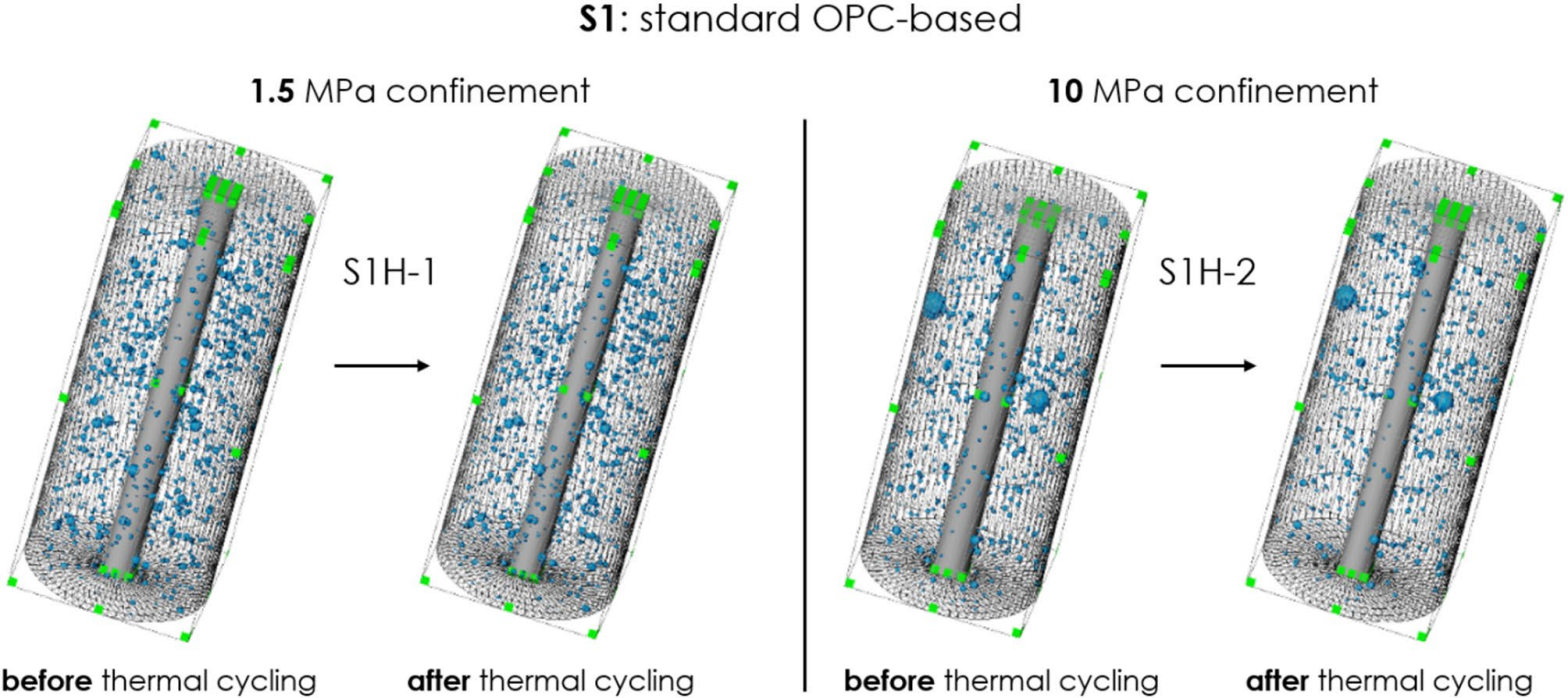
Microstructure of samples S1H-1(left, under 1.5 MPa confinement) and S1H-2 (right, under 10 MPa confinement) before and after thermal cycling (standard OPC). Voxel resolution of 32 µm. No cracks or significant alterations are observed in either sample following thermal treatment.

**Fig. 4:**
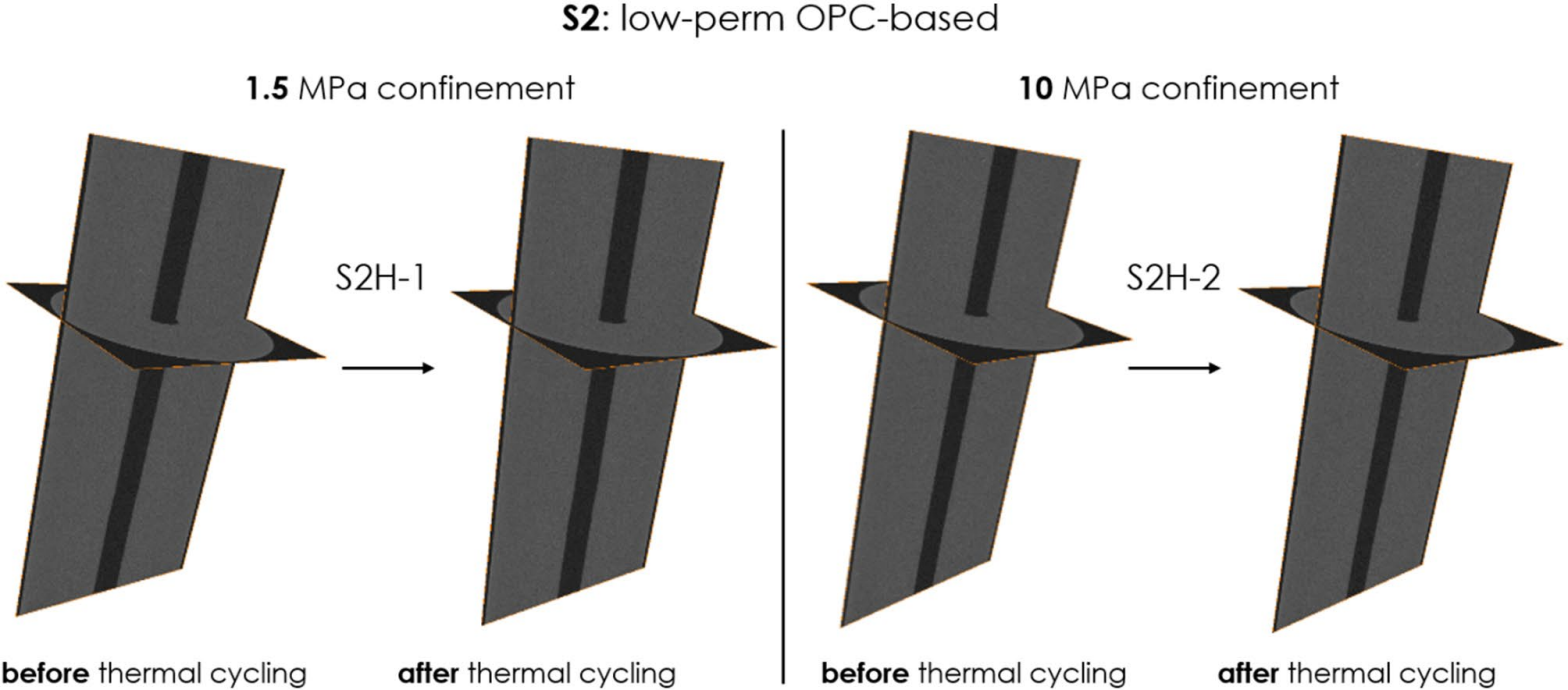
Microstructure of samples S2H-1(left, under 1.5 MPa confinement) and S2H-2 (right, under 10 MPa confinement) before and after thermal cycling (ultra-low permeability OPC-based). Voxel resolution of 32 µm. Black areas are for low density, and thus no cracks or significant alterations are observed in either sample following thermal treatment.

**Fig. 5:**
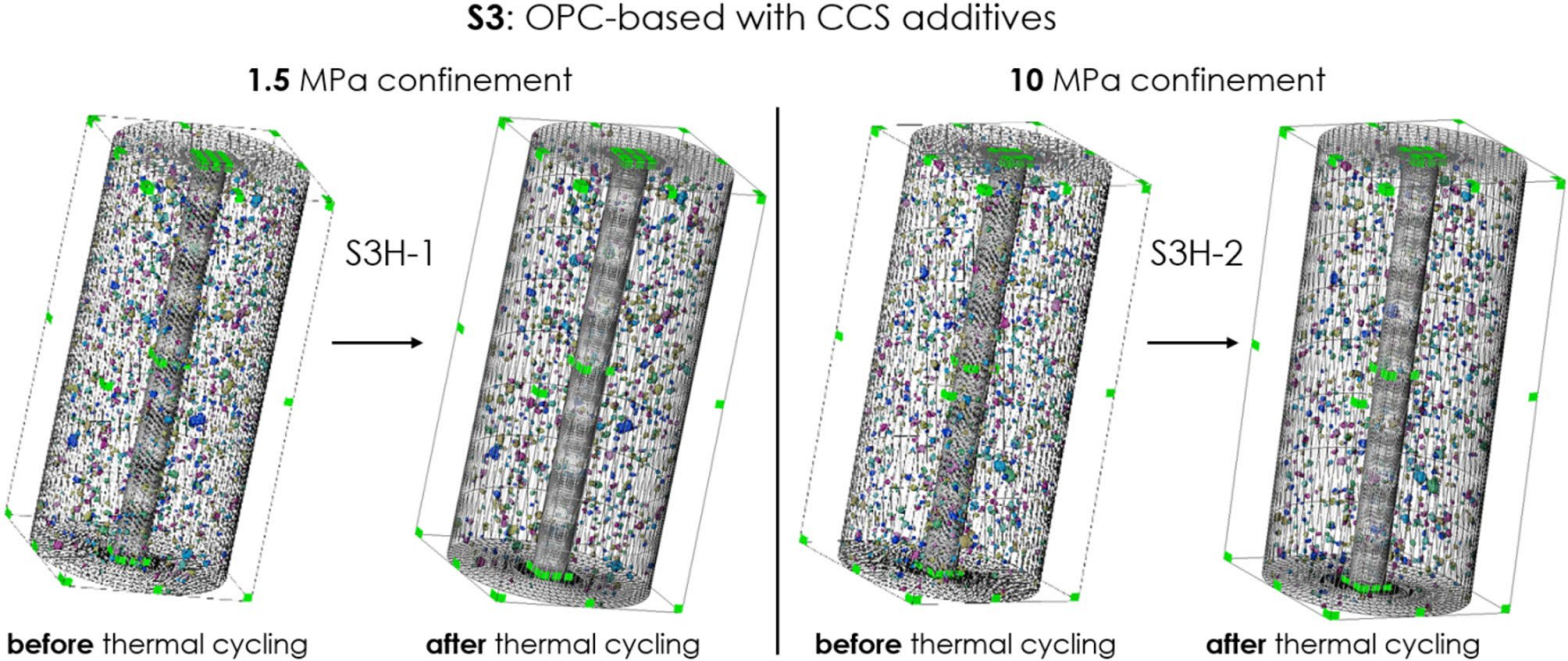
Microstructure of samples S3H-1(left, under 1.5 MPa confinement) and S3H-2 (right, under 10 MPa confinement) before and after thermal cycling (OPC-based with CCS additives). Voxel resolution of 32 µm. No cracks or significant alterations are observed in either sample following thermal treatment.

**Fig. 6:**
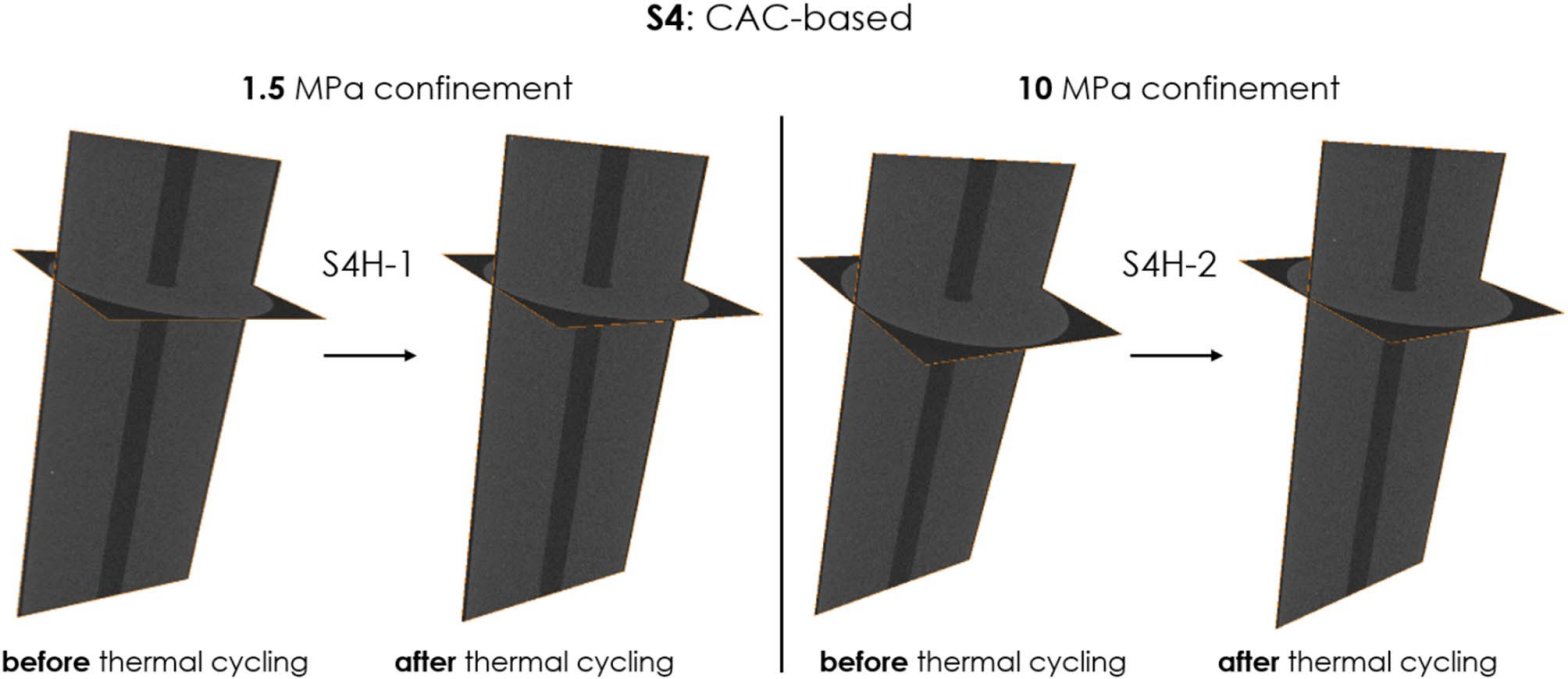
Microstructure of samples S4H-1(left, under 1.5 MPa confinement) and S4H-2 (right, under 10 MPa confinement) before and after thermal cycling (CAC-based). Voxel resolution of 32 µm. Black areas are for low density, and thus no cracks or significant alterations are observed in either sample following thermal treatment.

Figures 3, 4, 5, 6 show the microstructure of the two samples obtained with CT-scans, one under 1.5 MPa confinement and another under 10 MPa confinement, before and after thermal-cycling experiments for all tested sealant compositions (S1 to S4). Only samples of composition of S1 or S3 include many pre-existing voids, also before thermal treatment (Figs. 3 and 5). For samples of composition S2 and S4 orthogonal slices are shown in Figs. 4 and 6, since these compositions do not have pre-existing voids.

As the figures show, there are no visible cracks induced through thermal cycling in any of the sealants tested. Table 4 shows the overall void volume in the sample before and after thermal treatment ($V_{0}$ and $V_{TC}$, respectively, based on CT scans), the percentage decrease in void volume, porosity by micro-CT of the intact sample ($\phi_{CT}^{0}$), the porosity decrease by micro-CT ($\Delta \phi_{CT}$), and effective porosity measured by helium pycnometer.

**Table 4:** The overall void volume in the sample before and after thermal treatment ($V_{0}$ and $V_{TC}$, respectively), the percentage decrease in void volume, porosity by micro-CT of the intact sample ($\phi_{CT}^{0}$), the porosity decrease by micro-CT ($\Delta \phi_{CT}$), and effective porosity by helium pycnometer. Note that the voids, identifiable through micro-CT are larger than 32 µm.

Sample name	Sealant composition	Confinement [MPa]	Total volume of voids [mm^3^]			$\phi_{CT}^{0}$, [%]	$\Delta \phi_{CT} = \frac{{V_{0} - V_{TC} }}{{V_{sample} }}$, [%]	Effective porosity by helium pycnometer [%]
			$V_{0}$	$V_{TC}$	$\frac{{V_{0} - V_{TC} }}{{V_{0} }}$, [%]			
S1H-1	S1, OPC blend	1.5	201	192	4	0.406	0.02	35.6 ± 1.9
S1H-2		10	232	207	11	0.469	0.05	
S3H-1	S3, OPC blend with CO_2_-sequestering additives	1.5	158	146	8	0.319	0.02	42.2 ± 0.9
S3H-2		10	141	114	19	0.285	0.05	

The data presented in this table pertains only to S1 and S3 samples, as S2 and S4 samples are densely packed and exhibit no discernible voids before and after thermal treatment.

For sealants S1 and S3, there is a reduction in void volume, indicating compaction after thermal cycling under either 1.5 MPa or 10 MPa confinement. The compacting effect is more noticeable under higher confinement at 10 MPa, leading to a greater decrease in porosity as observed by micro-CT. Note that the porosity determined by our micro-CT (32 µm/voxel) is significantly lower than the effective porosity measured by the helium pycnometer (see Table 2). This difference likely arises from the fact that pores responsible for the majority of the effective porosity of hardened cementitious materials, such as our sealants, exist below the microscopic scale^34,35^ and therefore cannot be detected by our micro-CT.

**Fig. 7:**
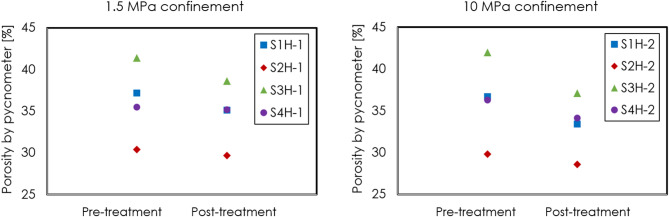
Effective porosity of samples from all four sealants before and after thermal treatment under 1.5 MPa (left) and 10 MPa confinement (right), as measured using helium pycnometer.

Figure 7 illustrates the effective porosity of samples of all four sealants before and after thermal treatment under 1.5 MPa (left) and 10 MPa confinement (right), as measured using helium pycnometer. Compared to the porosities determined by micro-CT, the porosities measured by pycnometer for all sealant samples are much higher and exhibit more notable decrease following thermal-cycling experiments under either 1.5 MPa or 10 MPa confinement. Particularly, under a higher confinement of 10 MPa, the reduction in porosities is more pronounced for all four sealants. The combination of visible micro-CT porosities and effective porosities by pycnometer corroborates that the thermal cycling hasn’t compromised the integrity of the sealant in our experiments conducted under confinement. On the other hand, these results do indicate that the application of a confinement leads to compaction of the sealants, with greater compacting effects observed at higher confinement.

### Effects of thermal cycling on circumferential strain and UCS on sealant samples under confinement

**Fig. 8:**
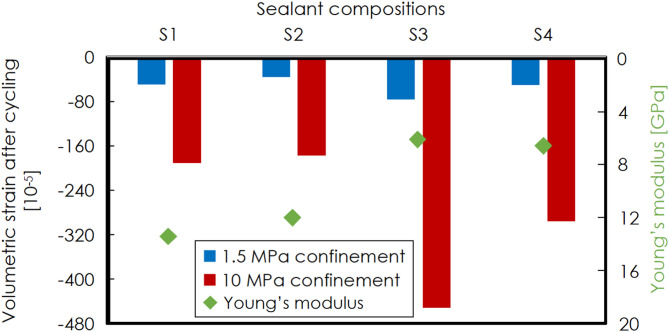
Bulk volumetric strain for all four sealants after thermal treatment under 1.5 MPa (blue) and 10 MPa confinement (red) and Young’s modulus (green) of four sealants. Note negative values indicate compaction here.

Figure 8 shows bulk volumetric strain of samples from all four sealants after thermal treatment under 1.5 MPa and 10 MPa confinement, based on the data obtained by the strain gauges inside the triaxial cell. All sealants show negative values of volumetric strain after experiments, which indicates sample volume reduction in our paper. For all sealants, sample experiences a greater reduction in volume after thermal treatment under 10 MPa confinement than after under 1.5 MPa. These reconfirm that confinement alone leads to compaction, with more pronounced effects at higher confinement. Compared to S1 and S2, S3 and S4 exhibit greater volume decrease after experiments, particularly under 10 MPa confinement. In addition, Fig. 8 displays Young’s modulus of four sealants. The differences in compactive strain for S3 and S4 compared to S1 and S2 correlate to the lower Young’s moduli of S3 and S4, compared to S1 and S2. With a lower Young’s modulus, sealants are more pliable and therefore compact more compared to stiffer sealants S1 and S2.

**Fig. 9:**
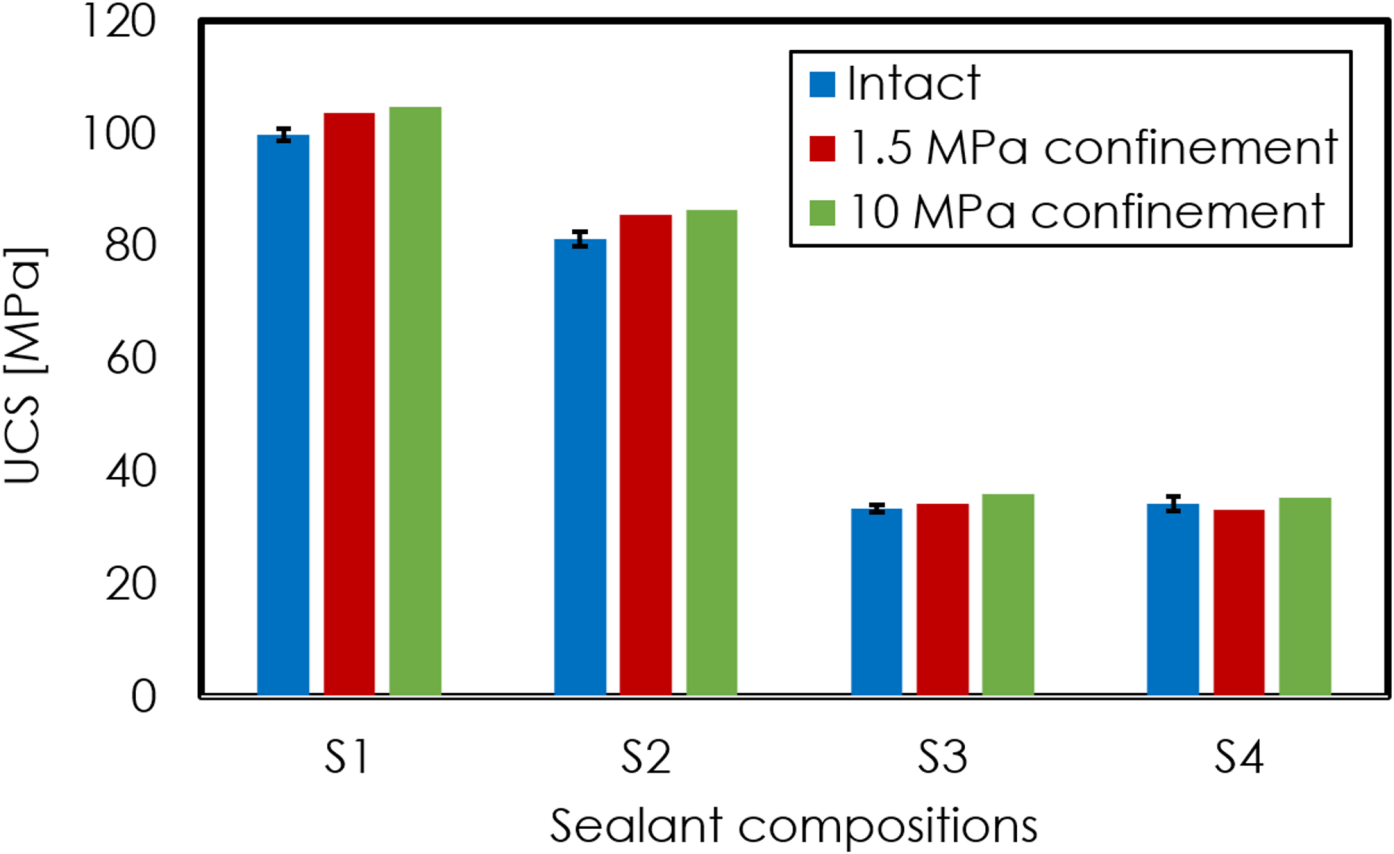
UCS of both intact samples and those subjected to thermal-cycling experiments under 1.5 MPa and 10 MPa confinement for four different sealants. Error bars for the untreated samples are based on measurements for three samples, each made at an interval of 1 month, and are presumed to be similar to the samples having undergone thermal treatment.

Figure 9 shows the unconfined compressive strength (UCS) of both intact samples and those subjected to thermal-cycling experiments under 1.5 MPa and 10 MPa confinement for four different sealants. Following thermal cycling under confinement, the UCS of samples from all four sealants demonstrates an increase, with a more pronounced enhancement under higher confinement. Note that for the S4 sample exposed at low confinement, a minor decrease in UCS is measured, though this may be due to sample variability.

## Discussion

**Fig. 10:**
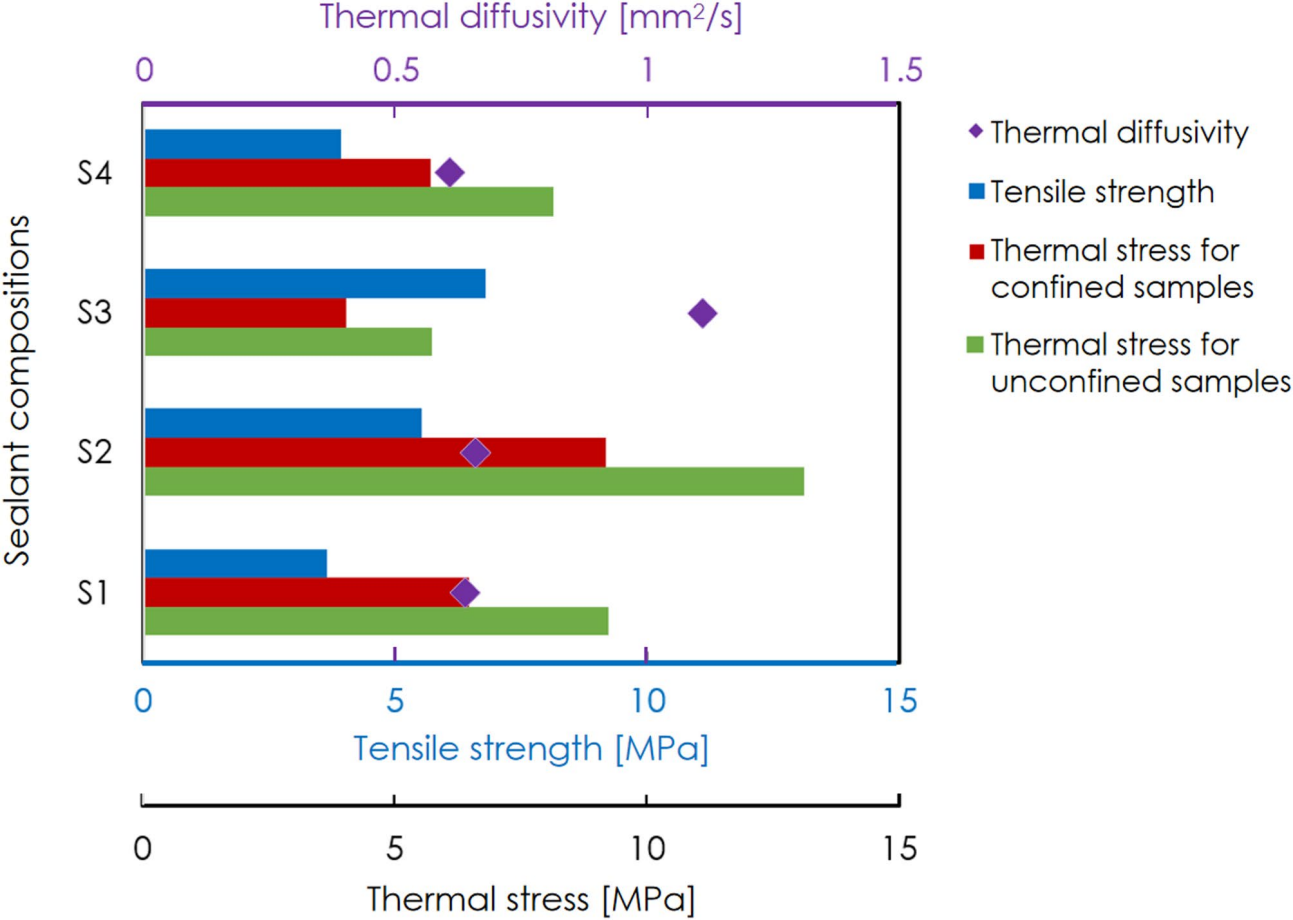
Thermal diffusivity and tensile strength of the four sealants, along with the thermal stresses induced by confined and unconfined thermal-cycling experiments on the four sealants. Thermal diffusivity and temperature changes used for thermal stress calculations in unconfined flow-through experiments are from Li and Pluymakers^26^. Note that tensile strengths are only measured on intact samples that have not undergone confinement or thermal cycling.

Figure 10 illustrates the tensile strength of the four sealants, along with the thermal stresses induced by confined (in this study) and unconfined^26^ flow-through thermal-cycling experiments on the four sealants. Thermal stresses are calculated according to Eq. 2. Note that thermal stresses referenced herein represent the local peak stress estimate in the vicinity of the borehole inlet of the sample (not a volume-averaged stress), which should coincide with the location of the most significant temperature drop, presumed here to be 70 °C for confined experiments, and 100 °C for unconfined experiments. This temperature range is within the upper range that can be expected for offshore locations, and therefore is a worst-case scenario. Note that the difference in thermal stresses between unconfined and confined experiments arises primarily from the difference in experimental procedure. There is a diminished temperature drop experienced under confinement during thermal cycling, since in the triaxial deformation setup (Fig. 1), the path that the injected water must traverse through the apparatus to reach the sample is relatively long, which results in its heating. Furthermore, during confined testing, the hot confinement medium surrounding the sample may have buffered temperature changes at the sample surface. Despite this constraint, thermal stresses induced by confined thermal cycling remain greater than the tensile strength for all sealants except S3, whereas those from unconfined thermal cycling are even more pronounced. Additionally, thermal diffusivity determined by Li and Pluymakers^26^ is also in Fig. 10, reflecting the transient heat transfer rate within the sealants. Sealants with higher diffusivity exhibit more efficient heat conduction, which results in a relatively lower loading rates of the thermal stress.

**Fig. 11:**
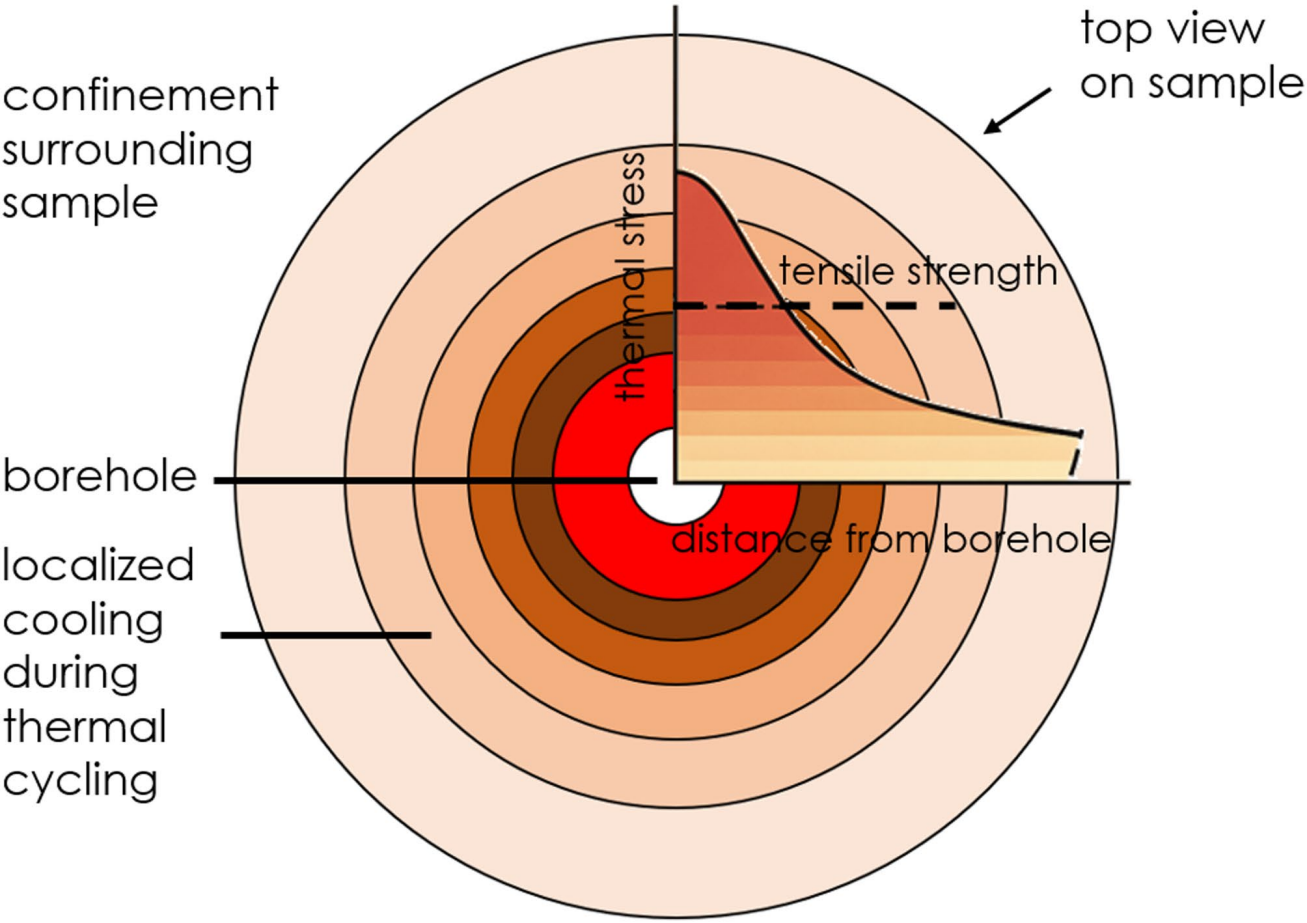
Schematic of thermal stress distribution throughout the sample. Darker colour indicates higher thermal stress-accumulated zones which are in vicinity of the borehole of the sample, and lighter colour indicates lower thermal stress-accumulated zones which are further away from the borehole. Note that the profile of the thermal stress vs. tensile strength vs. the distance from the borehole is conceptual.

In experiments without confinement, thermal stresses surpass the tensile strength. This was considered to be the key cause for the loss of integrity and emergence of thermally-induced mode I cracks^36^ in samples of all sealants except S3 (see Fig. 7 in Li and Pluymakers^26^). This mechanism of failure is consistent with previous studies on wellbore integrity under thermal loading, where tensile stresses from cool-down cycles are a primary driver for cement or wellbore damage^25,37^. In these unconfined tests, the formation of the mode I cracks predominantly originates from tensile stresses incurred during the rapid cooling phases of the samples. As shown in Fig. 11, during abrupt cooling, the heat concentrated near the borehole dissipates rapidly into the cold water within the borehole, resulting in a sharp temperature decline and subsequent shrinkage in that vicinity, thereby initiating thermal stresses. Regions further from the borehole undergo a delayed reaction, experiencing a lower temperature drop and correspondingly, less shrinkage and lower thermal stress. This creates a steep stress gradient, a classic precursor to thermal shock failure in brittle materials^38^. As thermal stresses induced by this thermomechanical deformation propagate temporally and spatially throughout the sample, they oppose the tensile strength of the sealants. The exceptional performance of sealant S3 can be attributed to its comparatively high tensile strength and thermal diffusivity, and its low susceptibility to thermal stress accumulation (calculated according to Eq. 2), because of its lower Young’s modulus and thermal expansion coefficient compared to the other sealants tested (see Table 2). All these favourable thermomechanical properties of sealant S3 enable it to withstand unconfined thermal cycling without compromising integrity.

In the current study, the lower thermal stress generated under confinement still exceeds the tensile strength for all sealants excluding S3. However, as confining pressure acts to inhibit tensile failure^39^, the initiation and propagation of cracks would require the thermally-induced stress to overcome the sum of tensile strength and confining pressure. In the confined configuration, the cooling effect is spatially localized around the borehole, while surrounding material and the confining fluid buffer temperature changes, resulting in a heterogeneous stress distribution. Consequently, the spatial extent of critical stress zones is limited, which also likely prevents crack initiation and propagation throughout the sample. In addition, as shown through UCS measurements, confinement also acts to enhance the mechanical strength of our samples, likely through closure of voids and pores in the samples. For all samples, a confining pressure of 10 MPa exceeds the generated maximum thermal stress, and is thus clearly sufficient to inhibit any crack generation/propagation. However, for samples S1, S2 and S4, a confining pressure of 1.5 MPa is insufficient to inhibit tensile cracking induced by thermal stress, as the maximum thermal stress generated exceeds the sum of tensile strength and confining pressure implying that under these conditions thermal cycling should have induced cracks. That no such cracks are observed may be explained by the limited spatial extent of the worst-case thermal stresses presented in Fig. 10, as peak thermal stresses steeply decrease moving away from the injection point both axially and radially. As a result, the generation of tensile stresses due to thermal effects is too limited spatially, to induce crack formation visible in CT-scanning. Therefore, the experimental results presented here and in^26^ clearly show that confinement exerts a strong control over the thermomechanical behavior of wellbore sealants, and act to inhibit fracturing when they are exposed to thermal cycling. This implies that, at greater CO_2_ storage depths where confinement is higher, the likelihood of sealant failure induced by thermomechanical cycles should be expected to be lower than at shallower depths.

In all our experiments, samples were completely dried before thermal treatment, and became slightly more water-saturated during the experimental procedure. While we took great care to dry our samples gently, drying may induce structural changes in cementitious materials that may affect mechanical behavior. To assess this, Li and Pluymakers^26^, who worked on the same materials in a similar procedure, performed controlled experiments to test the effects of wetting/drying cycles on similar sealant samples. Their results did not show any significant microstructural or mechanical effects of such cycles on these materials. Wellbore seals, especially those in wells drilled into deep (saline) aquifers, are typically assumed to be water-saturated, due to the presence of brine and other formation fluids^40,41^. Under such conditions, in addition to affecting the seal materials directly, thermal changes would also result in pore pressure fluctuations within the seals, especially when these pores are relatively isolated. Such fluctuations in pore pressure may provide an additional mechanism for the initiation and propagation of microcracks in seals exposed to thermal cycling. However, in our current experiments, we chose to focus on using dried sealant samples, in order to focus our research on how the thermal properties and the mechanical properties of these sealants are linked. A better understanding of this correlation will aid in well-design and in understanding the type of tests needed to determine if sealants are suitable for CCS applications. As a result, our work does explicitly disregard any issues related to pore fluid pressure build up in drained vs. undrained material, and we recommend that this should be addressed in future research. Note also that location-specific temperature and pore pressure gradients can lead to variations in water saturation within the wellbore, where it may fluctuate below full saturation levels, especially at shallower depths, or where gas may be present^42^. Moreover, in CCS scenarios, continuous CO_2_ injection may lead to a dried-out zone, potentially 100 s of meters in lateral extent around the injection wellbore, where the formation water has been driven off and/or dissolved into the CO_2_^43^. Sealant material close to such an injection point may also dry out over time. Nevertheless, while the findings presented in this study with dried sealants provide critical insights into their thermomechanical behavior, future studies incorporating saturated conditions are essential to capture the coupled poromechanical effects and fully assess sealant integrity under realistic CCS scenarios.

We use water instead of CO_2_ as the injection fluid, focusing on the mechanical response of sealants to rapid temperature changes. This approach isolates thermally-induced mechanical stresses, allowing us to assess sealant integrity under CCS-relevant thermal cycling without the added complexity of CO_2_-specific chemical reactions. This simplification excludes factors: (1) chemical effects (acidification, carbonation) that alter cement properties over time; (2) wettability differences, as CO_2_ is non-wetting and affects capillary behavior and leakage risk; and (3) drying from CO_2_ exposure, which may influence shrinkage and cracking. As a result, the findings represent a lower-bound estimate of short-term thermomechanical damage, most relevant to early injection cycles or pre-carbonated, chemically stabilized regions.

While the timescales of thermal cycling in our laboratory experiments (hours) are significantly shorter than those expected in field CCS operations (months to years), the key processes governing sealant integrity, namely, the generation of thermal stresses from temperature gradients and the mechanical response of the material—are controlled by intrinsic material properties (thermal diffusivity, tensile strength, Young’s modulus) rather than the absolute duration of the cycle. In the field, cold CO_2_ injection creates thermal gradients at the wellbore interface, which may evolve more slowly but will still impose significant tensile stresses on the sealant material. Our experimental design accelerates these gradients to induce representative stress magnitudes within a manageable laboratory timeframe. Although this results in a higher frequency of cycling than would occur in-situ, it enables us to evaluate the susceptibility of sealants to thermally-induced damage mechanisms under bounding conditions. Therefore, while absolute time compression is inherent to laboratory feasibility, the findings on the role of confinement and thermomechanical properties in mitigating thermal stress-induced failure remain directly relevant for material performance assessment and design considerations in CCS applications.

In addition, the observed correlation between sealant thermomechanical properties and their resistance to thermal cycling-induced damage aligns with classical theories of thermal shock resistance in brittle materials. Hasselman^38^ introduced the “thermal stress crack stability” parameter, $R_{st}$, which integrates thermal conductivity, elastic modulus, thermal expansion coefficient, and material strength to predict thermal shock performance for ceramics. Consistent with the principles outlined by Hasselman^38^ our findings show that sealants with higher tensile strength and thermal diffusivity, coupled with lower Young’s modulus and thermal expansion coefficient, are better equipped to withstand thermomechanical cycling in CCS environments.

## Conclusions

We employed a triaxial deformation setup to determine how thermal cycling affects the integrity of four wellbore sealants with different thermomechanical properties whilst being under confining pressures of 1.5 MPa or 10 MPa (representative for CO_2_ storage depths). We tested two OPC-based sealants (S1 and S2) that have been in use for hydrocarbon production for several decades, as well as two novel blends designed specifically for the purpose of future CO_2_ storage (S3 and S4). S3 is OPC-based but contains CO_2_-sequestering crushed dunite, whereas S4 is a calcium aluminate cement (CAC). Note that Li and Pluymakers^26^ have previously studied the same four sealants as considered here using a very similar procedure but without confinement. Their key finding was that for three of the sealants tested, induced thermal stress exceeded the material’s tensile strength, resulting in failure of the samples. However, our study uniquely quantifies how confinement representative of CCS depths affects thermally-induced damage mechanisms in sealants. Our findings here reveal that, notwithstanding peak thermal stresses exceeding tensile strength for all sealants except S3, confinement effectively raises the threshold for crack initiation and therefore mitigates the unfavourable effects of thermal cycling on sealant integrity. This is not only because the confining pressure itself acts against the opening of thermally-induced cracks, but also because confinement additionally lead to compaction of existing pores and larger voids, enhancing the mechanical properties of the sealants (as seen in UCS measurements performed on thermally-treated and intact samples). The effect of confinement is more significant for higher confining pressure (10 MPa) than for lower one (1.5 MPa), implying that at greater storage depths, the risk of seal failure due to thermomechanical cycles should be smaller compared to shallower depths.

Overall, this study offers valuable insights into the future design and testing of sealants in the context of CCS where thermal cycling is expected, by elucidating the interplay between mechanical and thermal properties of sealants, and by underscoring the significance of considering confinement effects. Furthermore, our findings suggest that an ideal sealant candidate for enduring thermal cycling in CCS well environments should possess high tensile strength, high thermal diffusivity, and minimal susceptibility to thermal stress accumulation, indicated by low Young’s modulus and low thermal expansion coefficient.

## Data Availability

Data will be made available on reasonable request to Kai Li (kai.li@kit.edu).
